# Endoscopic diagnosis of esophageal fistula hidden below massive bleeding assisted by modified external cannula device

**DOI:** 10.1055/a-2155-3276

**Published:** 2023-10-06

**Authors:** Feifan Chen, Zhihan Wu, Kai Deng, Junchao Wu, Jinlin Yang

**Affiliations:** 1 Department of Gastroenterology and Hepatology, West China Hospital, Sichuan University, Chengdu, Sichuan, China; 2 Sichuan University-University of Oxford Huaxi Joint Centre for Gastrointestinal Cancer, Department of Gastroenterology & Hepatology, West China Hospital, Sichuan University, Chengdu, Sichuan, China

A 60-year-old man, with a history of surgery followed by chemoradiotherapy for cardiac cancer 5 years ago, was referred for backache and hematemesis. The abdominal computed tomography angiography (CTA) revealed a pseudoaneurysm of the descending thoracic aorta ( Fig. 1 ). Emergency endovascular repair was performed during which contrast media extravasated outside the descending aorta and flowed into the esophagus ( Fig. 2 ) before stent graft insertion. The endovascular intervention was successful, but bloody fluid was still drained from the stomach tube, together with hematochezia. A bedside gastroscopy was therefore arranged to detect ongoing or recurrent gastrointestinal bleeding.

**Fig. 1:**
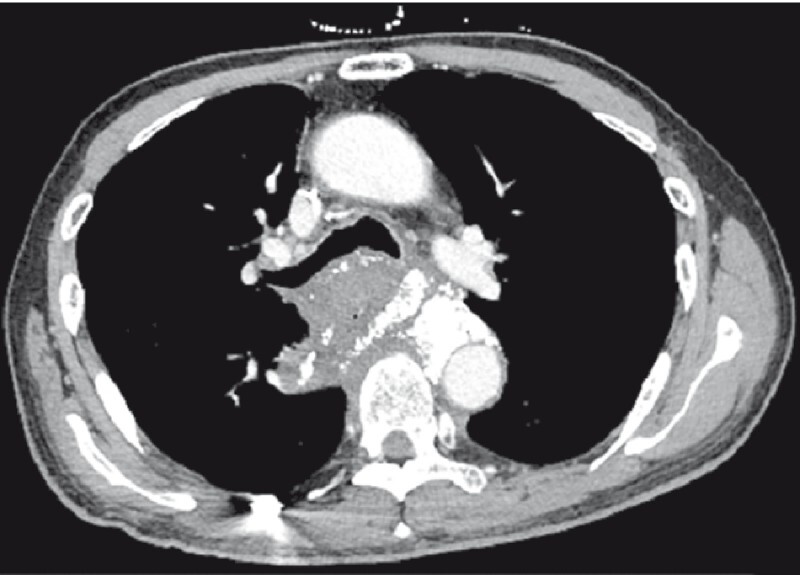
A pseudoaneurysm of the descending thoracic aorta on abdominal computed tomography angiography.

**Fig. 2:**
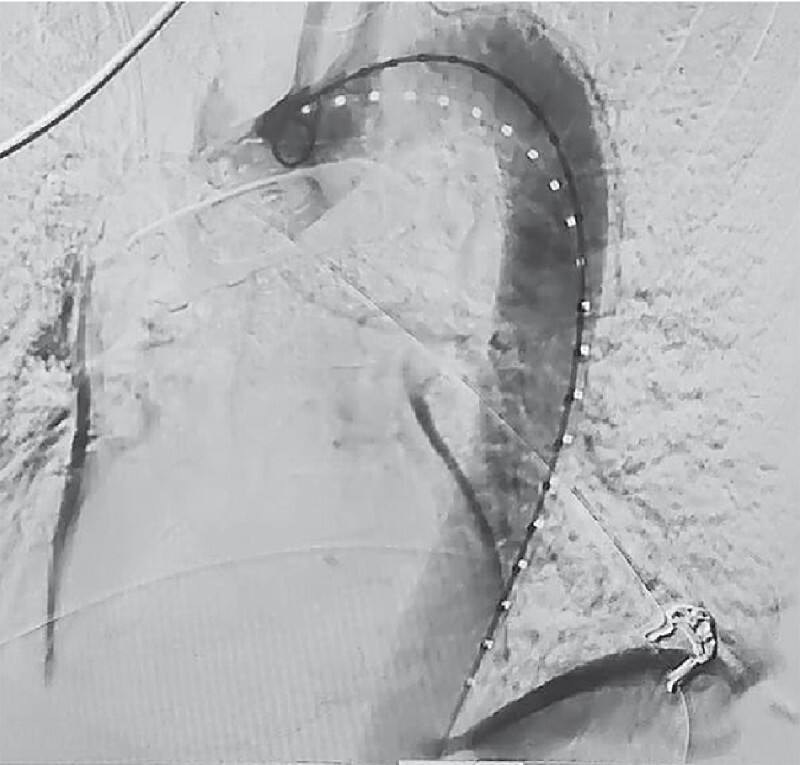
Contrast media extravasated outside the descending aorta and flowed into the esophagus before stent graft insertion.

Endoscopy initially revealed profuse bleeding and massive blood clots, making it hard to identify bleeding points ( Fig. 3 ). The modified external cannula was assembled and used to eliminate the clots ( Fig. 4 , Video 1 ) 1 . After suctioning and washing multiple times, the visual field was improved. The esophageal wall was rough and uneven with a patchy hemorrhage ( Fig. 3 ), and a huge fistula was spotted at 25 cm from the incisors, ruling out fatal bleeding ( Fig. 5 , Video 1 ). The whole procedure lasted for 10 minutes. Drugs, such as a proton pump inhibitor and hemostatics, were continued without a second surgery after discussion with surgeons. The bleeding then stopped with stable vital signs and hemoglobin, and the gastroscopy 5 days later showed no bleeding.

**Fig. 3:**
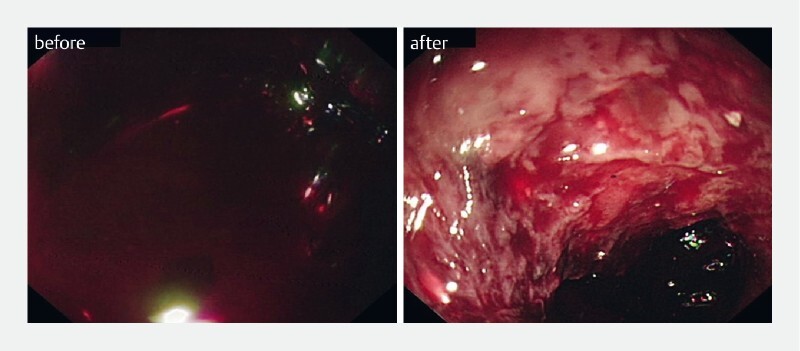
Endoscopic images before and after blood elimination with the help of the modified external cannula.

**Fig. 4:**
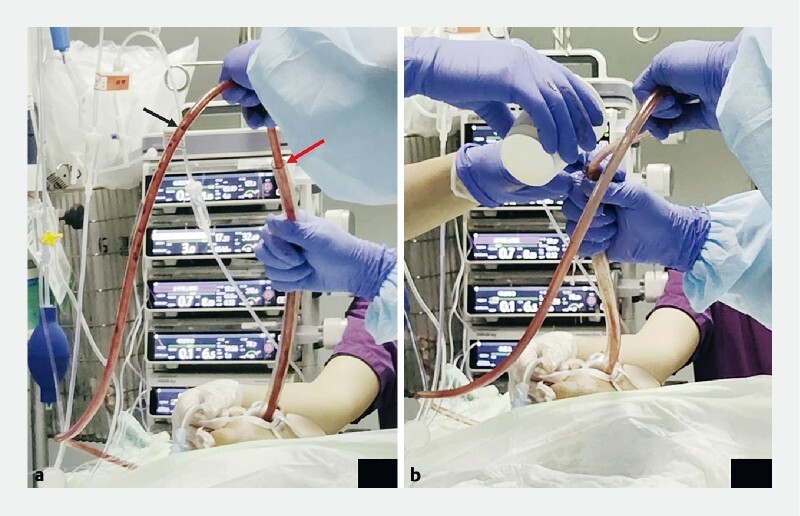
Procedure to clear away clots with an external cannula. **a** The suction tube with control pore (black arrow) was used to suction from the external cannula (red arrow). **b** Water was poured to facilitate the elimination of blood clots.

**Fig. 5:**
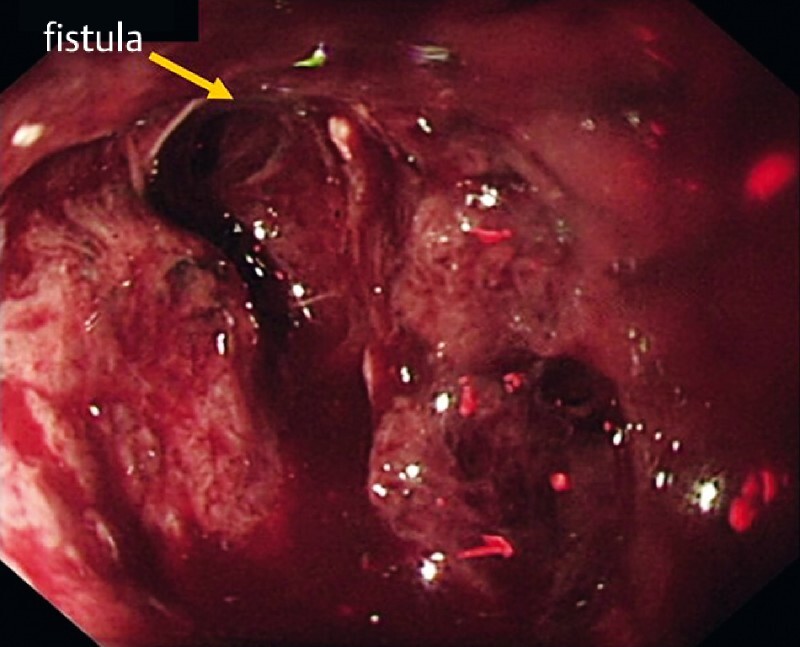
The esophageal fistula at 25 cm below the incisor (orange arrow).

**Video 1** Endoscopic diagnosis of esophageal fistula hidden below massive bleed assisted by modified external cannula device.


The modified external cannula is an innovative device for efficient removal of massive blood clots, especially during bedside endoscopic hemostasis, and has already shown great clinical value 1 . In this case, persistent bleeding significantly hindered endoscopic assessment, which was resolved by the modified device. The esophageal mucosa was cleaned and the fistula emerged, ruling out major bleeding and avoiding a second surgery. The additional diagnostic potential of the external cannula was fully realized, which greatly adds to its clinical benefit.

Endoscopy_UCTN_Code_CCL_1AB_2AZ_3AD

## Notes

Endoscopy E-Videos

https://eref.thieme.de/e-videos
*E-Videos* is an open access online section of the journal *Endoscopy* , reporting on interesting cases and new techniques in gastroenterological endoscopy. All papers include a high-quality video and are published with a Creative Commons CC-BY license. Endoscopy E-Videos qualify for HINARI discounts and waivers and eligibility is automatically checked during the submission process. We grant 100% waivers to articles whose corresponding authors are based in Group A countries and 50% waivers to those who are based in Group B countries as classified by Research4Life (see: https://www.research4life.org/access/eligibility/ ). This section has its own submission website at https://mc.manuscriptcentral.com/e-videos

## References

[1] Xiao X Yan H Liu J Novel approach for efficient removal of massive blood clots during emergency endoscopic hemostasis Endoscopy 2023 55 E248 E250 36427506 10.1055/a-1956-9244 PMC9831759

